# Inclusivity of Persons With Disabilities in the Work Sector During the Covid-19 Pandemic in Indonesia

**DOI:** 10.3389/fpubh.2022.835552

**Published:** 2022-03-29

**Authors:** Dumilah Ayuningtyas, Niken Sasanti Ardi, Sandra Barinda, Ayudina Larasanti, Theresa Napitupulu, Cindy Margaretha, Sahfira Ulfa Hasibuan

**Affiliations:** Department of Health Policy and Administration, Faculty of Public Health, Universitas Indonesia, Depok, Indonesia

**Keywords:** disability, inclusivity, workers, pandemic, Indonesia

## Abstract

**Introduction:**

The COVID-19 pandemic has had a significant impact on all levels of society, including people with disabilities, who in the pre-pandemic period faced obstacles in various sectors of life that affected efforts to fulfill basic living needs due to difficulties in accessing employment.

**Objective:**

The objective of this study was to identify various factors and causal interaction patterns that affect the inclusiveness of workers with disabilities in pandemic situations, a dynamic system is needed to capture causal interaction patterns related to the inclusiveness of workers with disabilities in pandemic situations.

**Method:**

This study used a causal loop diagram approach, which is part of a dynamic system that begins with determining the interaction of causal variables. The process of identifying and extracting data was carried out through a literature review and in-depth interviews with informants who met the principles of appropriateness and adequacy criteria.

**Result:**

The interaction pattern between the factors that influence the inclusiveness of disabled workers was depicted in three causal loop diagrams covering three major domains, namely social, educational, and economic aspects. The three causal loop diagrams showed an increasingly dynamic interaction pattern during the COVID-19 pandemic, considering that workers with disabilities have greater vulnerability, which impacts their level of acceptance and inclusiveness at work.

**Recommendation:**

There needs to be a specific policy to expand the acceptance of workers with disabilities by strengthening cross-sectoral collaboration and company commitments. The existence of a policy that prioritizes education, increases the budget, and procures adequate infrastructure for people with disabilities is a government commitment that is demanded to be fulfilled during the COVID-19 pandemic.

## Introduction

A disability is defined as a condition or function judged to be significantly impaired relative to the usual standard of an individual or group. The term refers to individual functioning, including physical impairment, sensory impairment, cognitive impairment, intellectual impairment, mental illness, and various types of chronic disease (1). People with disabilities (PwDs) are people who experience physical, intellectual, mental, and/or sensory limitations in the long term and who, when interacting with the environment, experience obstacles and difficulties in participating fully and effectively with other citizens based on equal rights (2). The inability and limitations experienced by PwDs often become obstacles to carrying out their daily activities, including getting a job. The stigma and community paradigms on PwDs often compare them to individuals who are unable to do anything and only need medical help, so they do not need education and work (3). This does not follow the regulations in the law, which state that every Indonesian citizen has the right to a worthy job (4). In addition to education, comfort, and welfare, work is essential for people with disabilities (5). Therefore, providing employment opportunities for PwDs is a challenge that needs to be considered by both the government and the community.

Prior to 1997, Indonesia issued regulations relating to PwDs, which stated that every person with a disability had equal access to work. Article 15 required that companies, both public and private, provide equal opportunities and treatment for PwDs by providing jobs according to the type and degree of disability, seen from the ability, education, and the amount that is adjusted to the company's qualifications (6). The regulation also stated that companies must employ at least one person with a disability who meets the requirements and qualifications for every 100 other employees. However, the reality is that few companies employ PwDs meet this minimum requirement (7–11).

In 2016, the Government of the Republic of Indonesia issued a new regulation regarding the number of disabled workers in a company, namely that government, regional, and state-owned companies employ a minimum of 2% of all employees with disabilities. Private companies are required to employ a minimum of 1% of the total number of employees (12). Based on data from Indonesian Statistics (BPS), the survey results show that the estimated number of PwDs in the labor market is 12.15%, with the medium category 10.29% and the severe category 1.87% based on the degree/severity of disability (13). In 2017, the Minister of Manpower stated that out of 440 companies with 237,000 employees, only 2,851, or around 1.2% of PwDs, were absorbed in the formal employment sector (14). Compared to the 2017 data, the number of disabled workers increased by 4,537 people in 2018 (15). In addition, companies that provide transportation facilities for workers with disabilities are still limited; five companies (7.04%) provide this service, while the remaining 66 companies (92.06%) do not provide transportation facilities for workers with disabilities (16).

The Covid-19 pandemic had a significant impact on all levels of society, including PwDs. PwDs also experienced obstacles in access, had difficulty meeting basic living needs, and were more vulnerable to Covid-19. There are several reasons PwDs are more at risk of contracting Covid-19, including barriers to implementing basic hygiene measures, difficulty in maintaining social distancing, depending on assistance in the form of touch from others (e.g., blind people), and limited information (17). For this reason, PwDs had to pay attention to their living conditions, as they were affected by the pandemic (18). The government needs to increase its support and attention to PwDs. Unfortunately, social services and rehabilitation of persons with disabilities program through the Ministry of Social Affairs is still limited, with only 19% of the budget available in 2010 (19).

The standard of living of PwDs needs to be increased through various government programs, not only related to the provision of regulations to increase the opportunities and competencies of PwDs at work, but also by increasing and expanding the education of PwDs. In this pandemic period, where online education is an alternative but with the limitations of PwDs, the support of the government, schools, communities, and families is needed (18). Unfortunately, government programs in the education sector also experienced delays during the pandemic.

This study aimed to identify various factors and patterns of interaction that affected the acceptance or inclusiveness of workers with disabilities during the Covid-19 pandemic using a dynamic systems approach.

## Materials and Methods

This study used a causal loop diagram approach, which is part of a dynamic system that begins by determining the interaction of causal variables. System dynamics involve mapping the system's behavior with the help of causal loop diagrams to understand the interdependencies between the parts of the system (20). Causal loop diagrams are a visual method that system thinkers use to explain feedback. The diagram is a language for articulating our understanding of the dynamic nature of the system being studied (21). The arrows indicate the direction of causality between cause and effect. The “+” symbol indicates unidirectional causality, and the “−” symbol in the arrow represents the opposite direction of causality.

Data were identified and extracted through a literature review and in-depth interviews. The research stage began with a literature review, which is the basis for researchers to identify factors and interaction patterns to build the initial causal loop design. Next, expert judgement was carried out to explore the literature review and sharpen the compiled causal loop diagrams. Expert judgment is the next stage in confirming the causal loop diagram and is performed by inviting an expert who has an educational and research background in the field of disabilities by in-depth interview.

### Exploration Strategy

The literature sources for this narrative review were obtained from the Pubmed and Wiley Online Library databases. The author conducted a PRISMA Protocol Search to select articles based on title, abstract, and full articles and to determine their suitability to the research topic. The search used a combination of the keywords “employee,” “disability,” “inclusivity,” and “pandemic.”

### Article Criteria

The inclusion criteria for this study were (1) written in English, (2) articles published from January 1, 2020 to September 7, 2021, (3) open access, (4) full-text articles, (5) all types of articles, and (6) contained keywords. The exclusion criteria were: (1) written in a language other than English, (2) article publications conducted before 2020, (3) not freely available, (4) incomplete article text, and (5) no keywords.

### Study Selection

Articles were selected based on the inclusion and exclusion criteria. Relevant and complete articles were included. The author chose articles based on the abstract and title of the article independently and was not bound by any party. The author made comparisons and decided on the similarities and differences between the selected articles. If there were any doubts about the abstract of an article, then the full text of the article was reviewed. A mutual agreement was made after each article was discussed.

**Figure 1:**
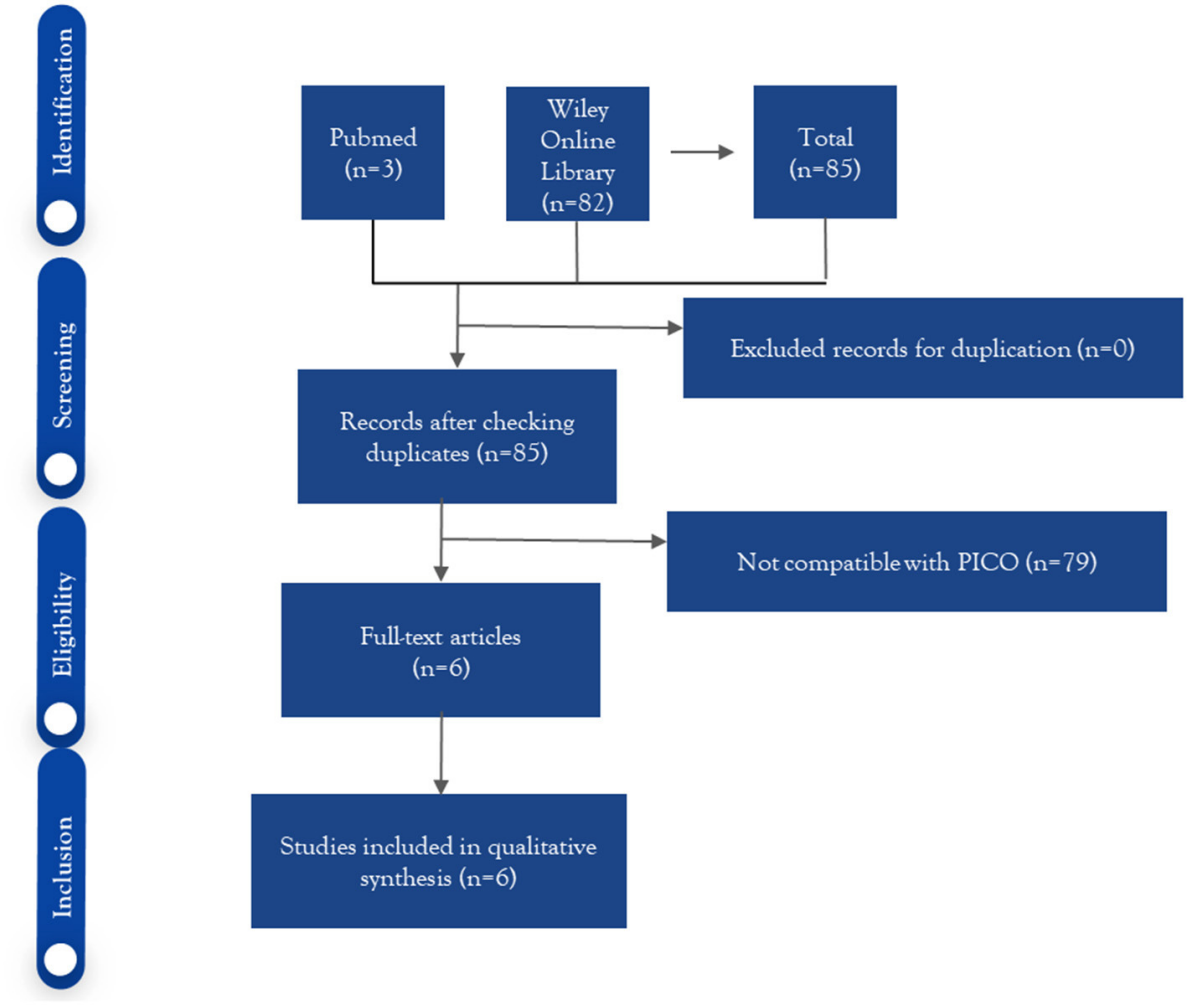
PRISMA protocol research.

Figure 1 shows the process of excluding research articles. The author obtained three articles from Pubmed and 82 articles from the Wiley Online Library. The authors had six articles remaining for eligibility selection, and all articles were selected for full article review because they fit the predetermined inclusion and exclusion criteria.

## Results

Based on the literature review results (see Table 1), the factors that influence the acceptance of disabled workers are socialization, cooperation, companies, PwD workers, PwD income, motivation, PwD unemployment, PwD careers, poverty, number of PwD students, education level, and PwD unemployment.

**Table 1:** Summary of literature review.

**No**	**Author/Location, Year**	**Title**	**Method**	**Summary**
1	Patricia Navas, Antonio M. Amor, Manuela Crespo, Zofia Wolowiec, Miguel A. Verdugo (Salamanca, Spain, 2020)	Supports for people with intellectual and developmental disabilities during the COVID-19 pandemic from their own perspective	Questionnaire	• Perspective: PwD • People who live in certain neighborhoods have less natural support, while those who live with families rely heavily on it. • Participants also lack the support they deem necessary. • People with disabilities also provide support to others. • Even though Pwd has received assistance during the lockdown, it must be ensured that proper support is provided wherever they live.
2	Lila Kossyvaki (Greece, 2021)	Autism education in Greece at the beginning of the 21st century: reviewing the literature	Scoping review	• Perspective: PwD Child, Family, teachers, society • Themes related to an autistic individual (i.e., the voice of autistic individuals and intervention studies), the family around them (i.e., the role of family and stigma), the role of teachers (i.e., teacher training and teacher stress levels), and the wider society (i.e., parent-teacher collaboration, the medical/deficit model of disability and inclusion) emerged from the review. • The above themes are discussed in the light of the cultural characteristics of Greece and the recent economic crisis the country underwent as well as similar findings from other European countries.
3	Laura Crane, Jade Davies, Anne Fritz, Sarah O'Brien, Alison Worsley, Anna Remington (England, 2021)	Autistic young people's experiences of transitioning to adulthood following the Children and Families Act 2014	Qualitative (online survey and/or interviews)	• Perspective: PwD • A sample of young people reported varied experiences regarding the help and support they received and how much of a say they had regarding the choices and support available to them. • The types of schooling they accessed played a role here: young people in mainstream schools highlighted particular challenges in accessing appropriate support. However, many young people in special schools said they felt well supported. • Parental advocacy was crucial for all young people. • The need for the development of general life and self-advocacy skills was apparent, however, especially in preparing young people for life after school. • Encouragingly, most of our participants were generally happy with their current situation, despite identifying several areas for further improvement. Overall, the results highlight the importance of listening to? And learning from? Autistic young people, throughout their educational journeys and especially as they transition to adulthood.
4	Simone Reinders, Marleen Dekker, Jean-Benoît Falisse (2021)	Inequalities in higher education in low- and middle-income countries: A scoping review of the literature	Scoping review	• Findings The review highlights key elements for policy-makers and researchers: (1) the financial lens alone is insufficient to understand and tackle inequalities since these are also shaped by human and other non-financial factors; (2) sociocultural constructs are central in explaining unequal outcomes; and (3) inequalities develop throughout one's life and need to be considered during higher education, but also before and after. The scope of inequalities is wide, and the literature offers a few ideas for short-term fixes, such as part-time and online education. • Finally, they should consider relevant contextual determinants of inequalities.
5	Robert A Moffitt, James P. Ziliak (US, 2020)	COVID-19 and the US Safety Net	Literature review	• Perspective: Society • This shows that the safety net response to employment losses in the COVID-19 pandemic largely consists of increased support from unemployment insurance and food assistance programmes and inadequate response compared with the magnitude of the downturn. • This study discusses options to reform social assistance in the United States to provide more robust income floors in economic downturns.
6	Amanda Coles, Doris Ruth Eikhof (Canada, 2021)	On the basis of risk: How screen executives' risk perceptions and practices drive gender inequality in directing	Qualitative	• Perspective: Society • Perceptions of employers/decision makers in the industrial world regarding the safety risks of persons with disabilities affect the inclusiveness of providing opportunities for persons with disabilities to work.

In-depth interview was conducted with resource people who are active in the health research center of one of the universities in Indonesia and who are observers of PwDs. Interviewees argued that the factors that influence the inclusiveness of PwDs in work include the following:

Positive factors that can contribute to the acceptance of PwDs.

1. High enthusiasm for work;
2. Increased awareness and understanding of human rights;
3. Zoning at each level for PwDs;
4. Indonesia already has a policy on disability;
5. Assistive technology to access various teachings;
6. The government provides rewards and awards to companies that employ PwDs and have excellent social responsibility.

Negative factors that can still be constraining:

1. Interest in achieving higher education is still low;
2. Access to information is not widely circulated in the general public;
3. PwDs feel reluctant and afraid of not being able to adapt;
4. Society needs to broaden education;
5. Pandemic;
6. The implementation and perspective of the community toward disability is still in the paradigm that PwDs are not productive people;
7. Few have access to scholarships because most PwDs in special schools are prepared for jobs that can generate immediate income;
8. Special schools are not ready to become inclusive because they are not well connected with the industry or appropriately facilitated so that competitiveness capacity for work has not been optimized;
9. The abilities and expectations are not the same between companies and PwDs;
10. Limited support from the government for companies to accept PwDs to work;
11. Lack of volunteers who can accompany college students and develop teaching methods that could influence the quality of students, including technical skills to work;
12. Lack of readiness of lecturers to teach PwDs;
13. Lack of peer treatment for accepting PwDs;
14. Types of disabilities with substandard intelligence should be provided with suitable jobs.

From the in-depth interview, information was obtained that since 2019, the Ministry of Education and Culture has prepared zoning with one inclusive school at all levels that can accept people with disabilities so that people with disabilities enter the world of education leading to higher education. Unfortunately, however, during the pandemic, there is no news yet.

**Figure 2:**
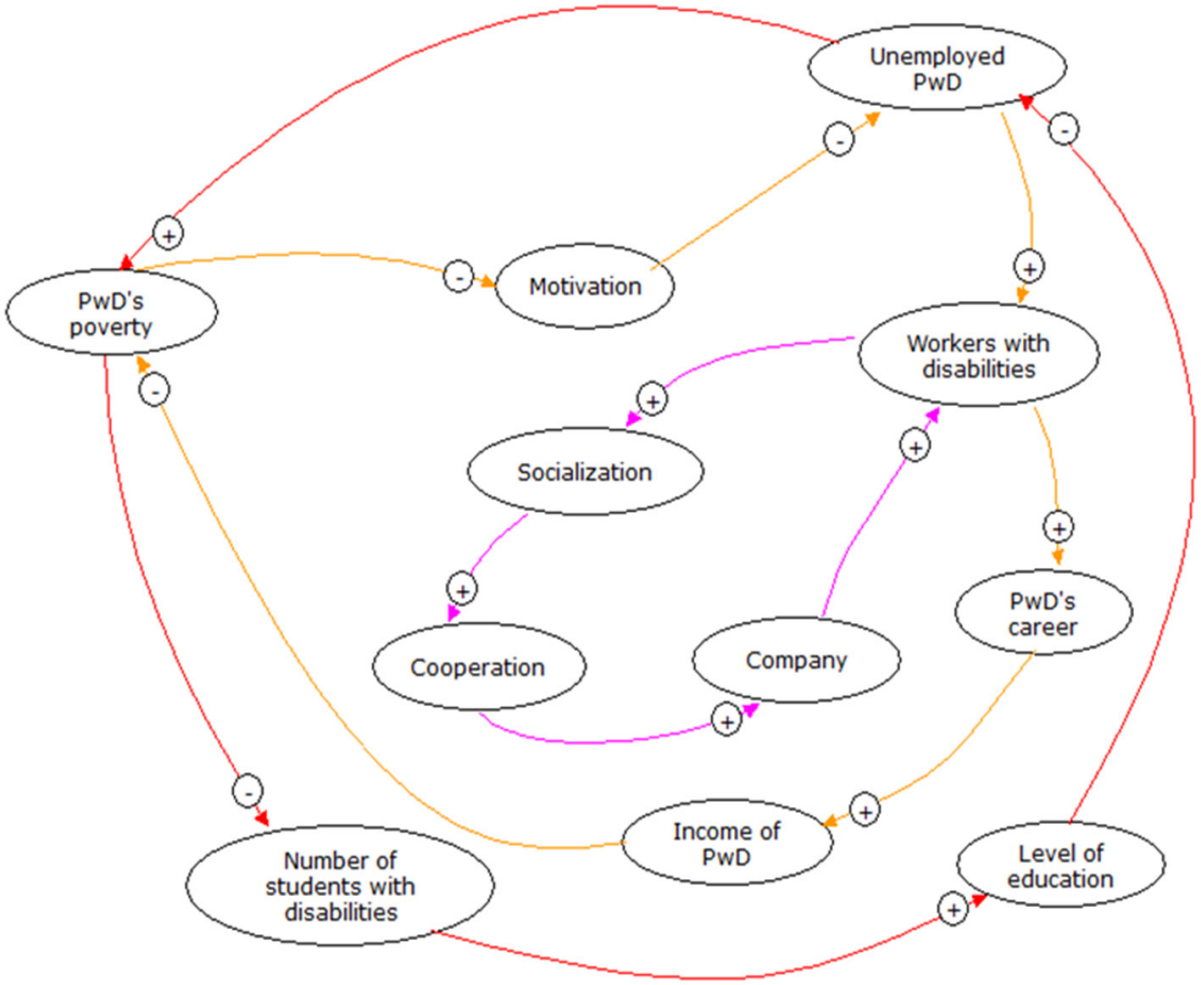
Casual loop inclusiveness of people with disabilities at work. The “+” symbol indicates unidirectional causality, and the “−” symbol in the arrow represents the opposite directions of causality.

The results obtained from the literature review, followed by in-depth interviews, showed the factors that influenced the inclusiveness of PwDs at work. The interactions of those factors are presented in the causal loop diagrams as follows (see Figure 2):

From the causal loop, three loops were identified. The three loops are seen in forming a cycle in the same direction, namely clockwise or counterclockwise.

### Loop 1

**Figure 3:**
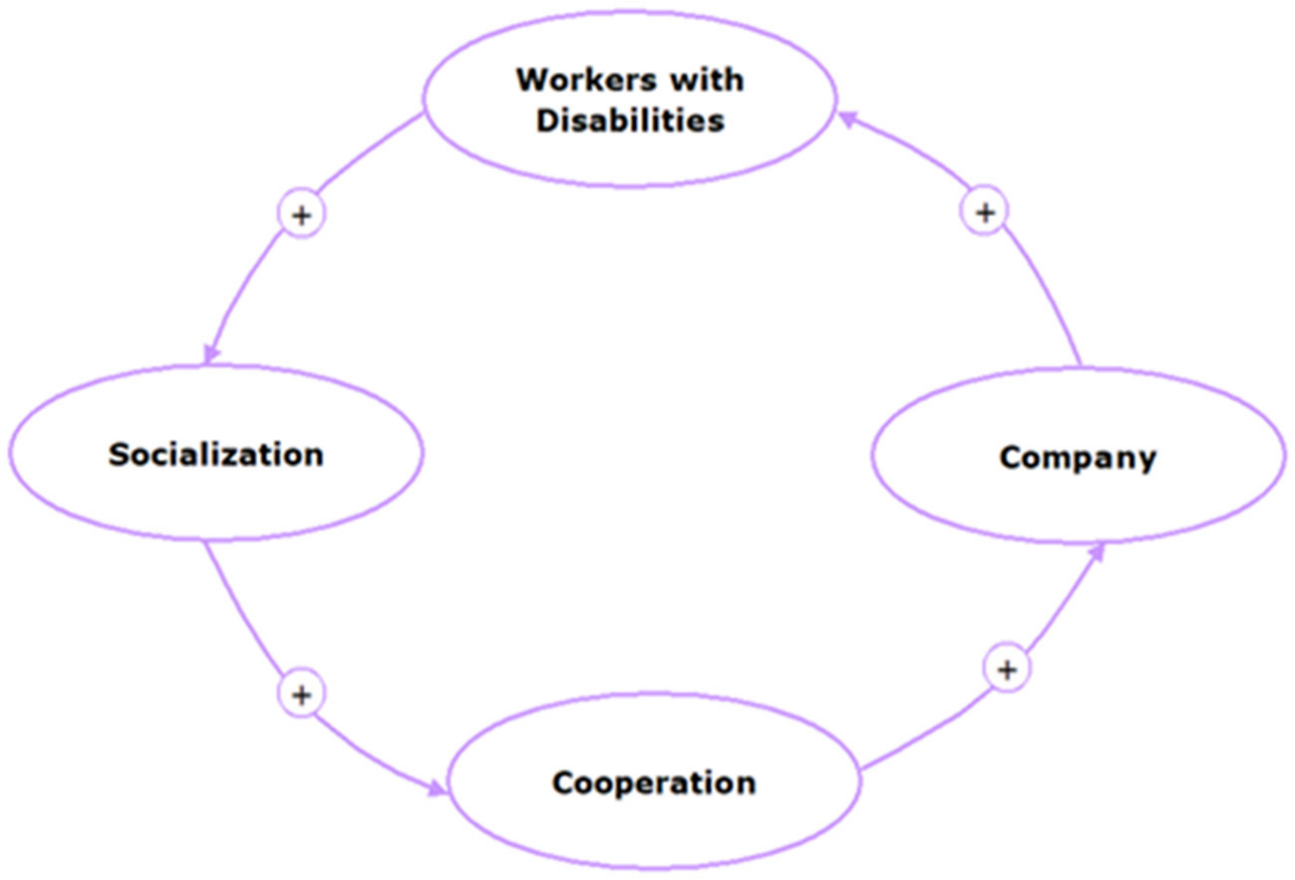
Loop 1: Socialization → cooperation → company → PwD → workers → socialization. The “+” symbol indicates unidirectional causality, and the “−” symbol in the arrow represents the opposite directions of causality.

Based on the Loop 1 (see Figure 3), socialization from the government regarding the policy to accept workers with disabilities in companies would increase cooperation between companies and companies with institutions and individuals who have a disability status. The involvement of workers with disabilities in a company can initiate other companies to accept workers with disabilities or indirectly socialize PwDs with particular abilities, showing their ability to contribute to a company.

### Loop 2

**Figure 4:**
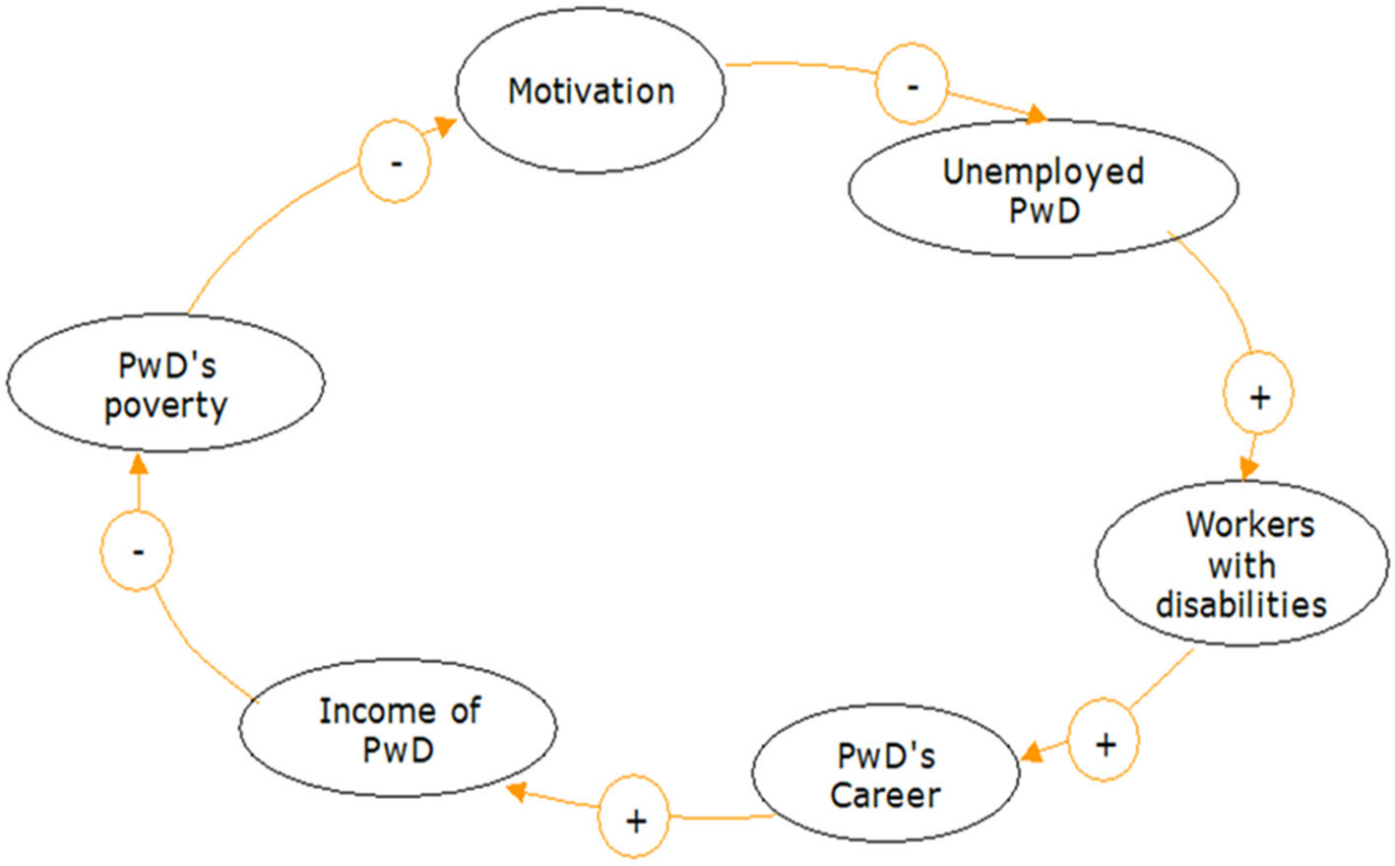
Loop 2: PwD income → Motivation → PwD unemployment → PwD workers → PwD careers → PwD income. The “+” symbol indicates unidirectional causality, and the “−” symbol in the arrow represents the opposite directions of causality.

In Loop 2 (see Figure 4), an increase in PwD's income also affects their motivation to get involved at work, which can reduce the high unemployment rate of PwDs. In other words, PwD's contribution to a company will increase, and they have the same right to a career to develop and earn an appropriate income.

### Loop 3

**Figure 5:**
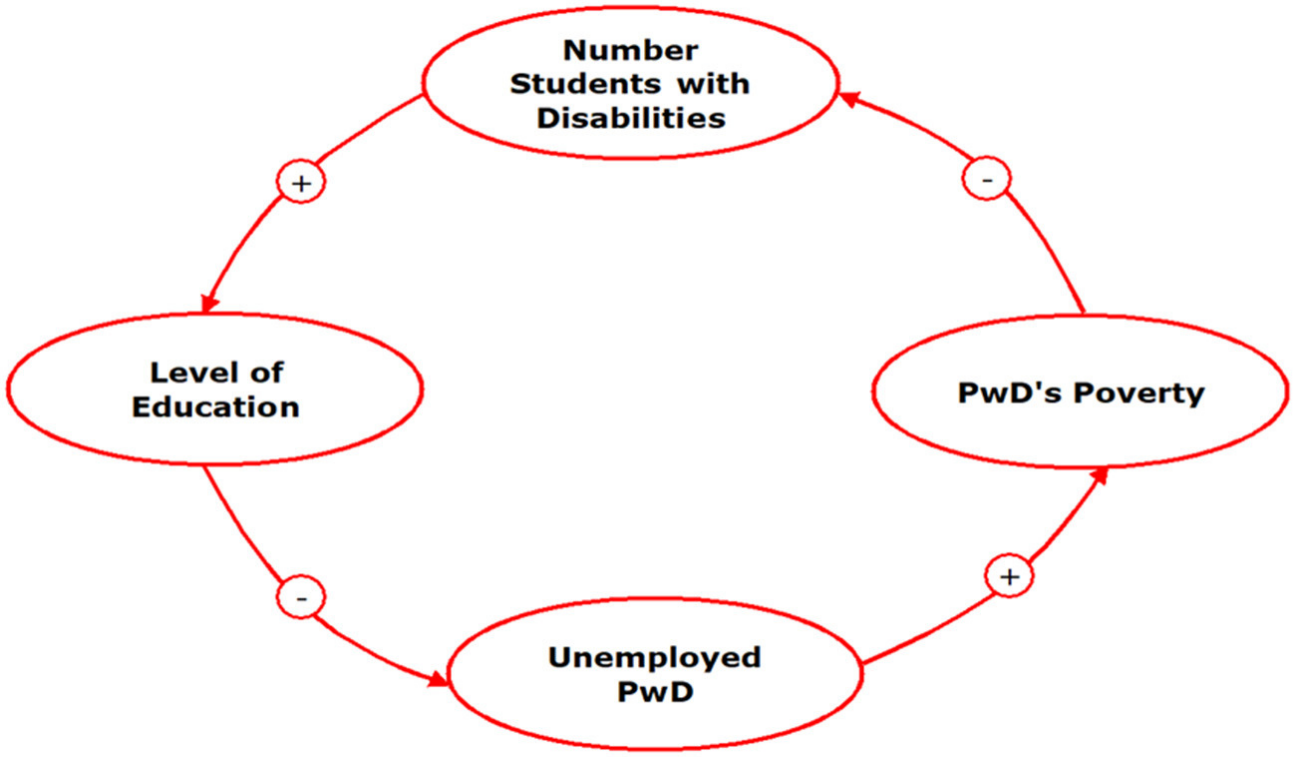
Loop 3: poverty → number of PwD students → education level → PwD unemployment → poverty. The “+” symbol indicates unidirectional causality, and the “−” symbol in the arrow represents the opposite directions of causality.

In Loop 3 (see Figure 5), PwDs with a history of low poverty rates determine the number of PwDs who can become students in education. Their easy access to education will increase their level of education. The level of education in PwDs will determine how much they are capable of and how they work, for example, reducing unemployment. A low PwD unemployment rate will increase income and reduce poverty.

## Discussion

### Loop 1: Socialization → Cooperation → Company → PwD Workers → Socialization

Through loop 1, socialization from the government regarding the regulation of employing PwDs will provide increased cooperation to government and non-government institutions. As in the data obtained in the 2015–2019 Rencana Aksi Nasional Hak Asasi Manusia/National Action Plan for Human Rights (RANHAM) related to the aspect of cooperation, there are at least 40 ministries/agencies and all provincial and district/city governments identified as institutions that have tasks, responsibilities, and roles related to improving the welfare of PwDs. In addition, there are five other ministries/institutions, namely Ministry of Youth and Sports, Ministry of Tourism, Ministry of Women's Empowerment and Child Protection, the Central Bureau of Statistics and the National Team for Acceleration Poverty Reduction (TNP2K), that have the potential to play a role but are not yet included in the RANHAM as stakeholders who fight for PwDs (3).

Based on the results of the Focus Group Discussion (FGD) SMERU Research Institute, the central government budget allocation for disability-related activities in 2017 was IDR 309 billion. The budget is mainly spread across the Ministry of Social Affairs, the Coordinating Ministry for Human Development and Culture, and the coordinating Ministry for Politics, Law, and Security. The distribution of the budget is uneven between ministries because around 90% is given to the Ministry of Social Affairs (3). This could be due to budget constraints and commitment to determining budget allocation priorities.

In addition, the environment, on the social aspect of humanity, which consists of donor agencies and non-governmental organizations or non-governmental organizations (NGOs), can adopt competitive or cooperative interactions between organizations. Collaboration between NGOs in providing assistance and information disclosure had a positive and direct effect on government policy (22). In other words, the more successful the government in each of its policies requires more cooperation between NGOs. With the strengthening of the level of NGO cooperation, the utility of organizations and donors increases, so this type of NGO interaction is also beneficial for donors. NGO competition provides the possibility of creating a higher level of social welfare with less budget consumption (22).

This collaboration can increase the perceptions of other job seekers about companies that employ PwDs. The social impact perspective is an inclusive practice that can be an example for other companies. A survey of various full-time employees across the US reported that job seekers tend to prefer inclusive employers, with approximately 80% of respondents identifying inclusivity as an important factor in choosing an employer (23). Research has also shown that consumers prefer companies that employ individuals with disabilities (24). Various beneficial results related to the presence of workers with disabilities provide confidence and the notion that workers with disabilities are an untapped resource or a hidden asset (24–26).

There was a relationship between good leadership and the level of discrimination, job satisfaction, and the need for recovery felt by disabled workers. In addition, good leaders display responsiveness to community calls, such as in terms of supporting PwDs, providing fair treatment, exploring potential, meeting the needs for psychological security, self-esteem, socializing, and changing negative views of themselves, and being able to turn them into employees with self-respect, confidence, and attachment (27).

However, attention to PwDs at work is still not well integrated. Not only does it require employment, but physical conditions that have limitations also require the support of facilities that can increase PwD mobility, such as public transportation and special aid. There were 71 business companies in six provinces in Java and Bali that employ PwDs, only about 7.04% of companies provide public transportation facilities for disabled workers (28). Furthermore, based on the results of a survey by Indonesia Corruption Watch (ICW) to 800 PwDs conducted randomly in four cities (Bandung, Solo, Makassar, and Kupang), only very few PwDs (9%) have ever received assistive devices. The majority (91%) of PwDs in Indonesia have never received assistive devices from the government (29).

The socialization of regulations to create employment opportunities for PwD workers could provide PwDs with a place to work and adjust the workload to the level of disability possessed by individual PwD workers. PwDs still have lower levels of work, job security, income, salary satisfaction, job satisfaction, and quality of work–life compared to people without disabilities (27).

### Loop 2: PwD Income → Motivation → PwD Unemployment → PwD Workers → PwD Careers → PwD Income

Motivation is a force that exists within and from outside a person and generates enthusiasm and perseverance (30). Work gives a person the opportunity to earn income, establish social relationships, and build socio-political status, which is also crucial for PwDs (31). Many PwDs are able and willing to work to be financially independent and contribute to the development of the wider community and society (32).

A career path is a path that connects one position to another. Career paths are needed both in private and public companies that are oriented toward work challenges. A company will provide and organize a work program to determine career paths to encourage employees to improve at work through a series of experiences and tasks that can be carried out in one or more organizations (33). Career path for PwDs must also be considered, as an employee's right in their work.

Career development involves two-way connections, namely vertical (e.g., responsibilities at work) and horizontal (e.g., existence of different competencies at the same level or job rotation). Both of these factors will affect the career development of each employee but also require additional special skills. Based on this, PwDs who work often feel that there is no justice in career development in companies with their physically limited abilities. It discriminates them from having access to jobs, and they are often given low-status jobs (34).

Companies are concerned about the limitations of the disability and generalize that to a PwD's ability to work. The existence of barriers to the career development of disabled workers will affect how they obtain a job and the income they receive. Opportunities to get promotions and job rotations are also limited (35).

The existence of limitations for PwDs causes them to be vulnerable to being classified as poor. The 2013 Riset Kesehatan Dasar/Basic Health Research Survey (Riskesdas) found that the prevalence of disability was higher at a lower ownership index. For example, 15.2% of PwDs are in the lowest wealth quintile index (4).

### Loop 3: Poverty → Number of PwD Students → Education Level → PwD Unemployment → Poverty

In Loop 3, it was shown that poverty can affect the ability of students with disabilities to attend school. Low-income families find it difficult to send their family members to school because of the family's economic limitations. Moreover, it could be that the main priority is not to get an education but to fulfill the family's basic needs, considering that the family's economy is very limited. This will undoubtedly affect the level of education for PwDs of school age. Furthermore, the low participation of PwDs in schooling in general will certainly affect their education level. For example, in Greece, it was reported that several autistic children did not have access to appropriate education or did not attend school (36, 37).

Several factors affect the level of education for PwDs:

#### Participation

Today's autistic individuals have limited involvement in their education and life decisions, let alone participation in research (38). From the author's personal experience of interviews with parents of children with autism in Greece, parents seemed very reluctant to let their children participate in the study. This reluctance may be the fear that with their children's participation, they will become aware of their autism diagnosis, which is often kept secret from them (38).

#### Family Role

The family, in its broad form, seems to play an essential role in the education of autistic children in Greece (38). The ability of parents to discipline their children is related to the progress of the child's development. Great family values should also be a concern, because not only parents but also grandparents and other relatives play an essential role in raising children in Greece (39).

#### Teacher's Role

Teaching children with autism has been found to cause considerable stress, even for special education teachers (40). It was extensively importance of parent-and-professional partnerships, especially in special needs individuals (41).

#### Stigmatization

One-third of parents do not disclose to their co-workers that they have an autistic child, mainly because they fear stigmatization or because this will affect their promotion opportunities (42). Fathers tend to be more secretive about having a child with autism than mothers. The extent of stigmatization of autistic individuals in Greek society found that several teachers believe that other students should not know about the presence of an autistic person (43).

#### Inclusive Education

Peer relationships play an essential role in the successful inclusion of students with autism in schools and the wider community (44). There is an interesting paradox about inclusion in Greece. On the other hand, studies show that teachers consider inclusion essential to minimize the impact of stigma on children with Special Educational Needs and Disabilities (SEND) and their families. On the other hand, many teachers think that students with autism may receive a better and more appropriate education in special schools (43, 45, 46).

Most Greek teachers thought that special schools seemed more suitable for them because they should receive social skills education rather than follow a curriculum that focuses on academic skills (47). The same thing happened in Indonesia. In principle, the curriculum is the same as the regular school curriculum, but in special schools it is more adapted to the abilities of the students. The basic competencies for children in special schools are more adapted to their disabilities, and each disability has different basic competencies (48). Teachers seem unclear about their responsibility to teach autistic children, especially in public schools. Many of them held onto some misconceptions about autism and stated that they needed more training. Teaching children with autism tends to increase teacher's stress levels, but appropriate training has been found to reduce their stress levels.

Inadequate education levels for PwDs will affect their acceptance of job searches, especially if they do not have sufficient skills. In addition, a limited level of education will reduce the bargaining power of the intended job location. In the end, the level of employment of PwDs will also be limited and will affect their economies in the future. The high unemployment rate causes problems in the economic and social sectors, such as poverty.

Living in poverty can increase the likelihood of students with disabilities becoming unemployed and having limited secondary education. The United States seeks to address educational problems and economic inequality for PwDs through federal legislation. Therefore, it is recommended that there be direct policies to address educational inequality for PwDs so that they are expected to improve their daily lives (49).

Studies have shown that poverty is related to employment in terms of disability. In a study in India, PwDs can only obtain an education if it is supported by accessible educational facilities, roads, and adequate transportation facilities and information. For that, both education and work require accessibility. These things will not happen without the support of adequate regulations and policies. To obtain these regulations, it is necessary to build awareness and good communication with the parties involved. Therefore, five things need to be considered simultaneously: employment, education, accessibility, regulation/policy, and good communication in India (50).

India has a 3% employment quota policy for PwDs, namely people with orthopedic, visual, and hearing disabilities. This policy can benefit PwDs in finding work. Although this policy has been established, in reality, it has not run optimally (50). The quota has not been fulfilled in full due to the limitations of PwDs who can fulfill the requirements. This quota may be fulfilled in the public sector, especially in government offices. However, for the private sector, a more effective regulation of employment without discrimination is needed. Overall, the main issues that need attention for the employment of PwDs are those with disabilities who do not receive education in schools, colleges, or universities and who are also not involved in skills development programs (50).

The Covid-19 pandemic has put all countries at the same starting point; no one is unaffected, and no government is ready to deal with it, especially in the economic field. It can be seen that the impact of the Covid-19 pandemic has also affected developed countries, such as the United States. The United States has social insurance programs for PwDs, such as disability insurance, Medicare, and additional security income. However, social insurance for PwDs does not assist those who are experiencing an economic downturn. The unemployment rate in the United States at the beginning of the pandemic rose sharply, especially during the Great Recession. During 2020, there was a decline in income for necessities. For this reason, it is necessary to reform options for US safety nets, especially for PwDs, including expanding program access and generosity to unprotected and unprotected populations during good and bad economic times, mainly through social assistance programs (51). In Indonesia, there was training for PwDs in the worker card program held by the government, so that PwDs will become more productive. As a result of the Covid-19 pandemic, there has been a reduction in wages, especially for PwDs with limited mobility. Therefore, with the training from the program, it will increase the value-added skills for PwDs. So the expansion of this program has become a necessity (52).

PwDs workers experienced reduced hours and experience higher levels of financial stress in the pandemic situation (53). Working-age adults with disability were particularly disadvantaged by the financial impact of the COVID-19 lockdown in the UK. This situation strengthens the study findings, namely the interaction between factors and causal interaction patterns that affect the inclusiveness of workers with disabilities in pandemic situations. This condition applies universally on an international scope until the World Health Organization (WHO) has indicated this. Disabled people experience entrenched structural disadvantages, including barriers to accessing health care, increased poverty, lower employment, and lower education levels compared to the general population (54–57).

Although the objectives of this study have been achieved (to identify various factors and causal interaction patterns that affect the inclusiveness of workers with disabilities in pandemic situations), we are aware of the limitations of our study that focus on causal loops and have not expanded studies with quantitative approaches to surveyed PwDs workers to explore their perceptions of those affected by the pandemic. For this reason, further research can be carried out using a quantitative analysis approach.

## Conclusion

1. The identified factors of inclusivity of people with disabilities in the work sector during the Covid-19 Pandemic are socialization, cooperation, company, PwD workers, PwD income, motivation, PwD unemployment, PwD career, poverty, number of PwD students, education level, and PwD unemployment.
2. Disability was proven to be significantly affected during the pandemic, impacting the job acceptance sector.

## Data Availability Statement

The original contributions presented in the study are included in the article/supplementary material, further inquiries can be directed to the corresponding author.

## Ethics Statement

This study was approved by the Research and Community Engagement Ethical Committee Faculty of Public Health Universitas Indonesia (Ethical Approval: Ket- 321/UN2.F10.D11/PPM.00.02/2020).

## Author Contributions

DA conceived the manuscript idea, supervised, and funded the article. DA, NA, SB, AL, and TN contributed to conception and design of the study and performed the causal loop. All authors performed the literature review, wrote the first draft of the manuscript, contributed to manuscript revision, read, and approved the submitted version.

## Funding

This study was funded by the Directorate of Research and Community Service, Universitas Indonesia, Indonesia, number: NKB-1620/UN2.RST/HKP.05.00/2020.

## Conflict of Interest

The authors declare that the research was conducted in the absence of any commercial or financial relationships that could be construed as a potential conflict of interest.

## Publisher's Note

All claims expressed in this article are solely those of the authors and do not necessarily represent those of their affiliated organizations, or those of the publisher, the editors and the reviewers. Any product that may be evaluated in this article, or claim that may be made by its manufacturer, is not guaranteed or endorsed by the publisher.
